# Impact of hospital volume on survival in patients with locally advanced colon cancer – A Dutch population‐based study

**DOI:** 10.1111/codi.17288

**Published:** 2025-01-26

**Authors:** L. C. F. de Nes, P. J. Tanis, R. H. Verhoeven, J. H. W. de Wilt, P. A. J. Vissers

**Affiliations:** ^1^ Department of Surgery Maasziekenhuis Pantein Boxmeer The Netherlands; ^2^ Department of Surgery Radboud University Medical Centre Nijmegen The Netherlands; ^3^ Department of Surgery Erasmus MC Cancer Institute Rotterdam The Netherlands; ^4^ Department of Research and Development Netherlands Comprehensive Cancer Organization Utrecht The Netherlands

**Keywords:** adjuvant therapy, hospital volume, locally advanced colon cancer, neoadjuvant therapy, surgical procedures, survival

## Abstract

**Aim:**

Locally advanced colon cancer (LACC) often necessitates complex prognosis‐determining treatment. This study investigated the impact of hospital volume on short‐ and long‐term outcomes following surgery for LACC.

**Method:**

Data involving all patients with LACC categorized as clinical T4 and/or N2, between 2015 and 2019 in the Netherlands, were extracted from the Netherlands Cancer Registry. Hospitals were stratified into low volume (1–19 LACC resections per year), medium volume (20–29 LACC resections per year) and high volume (≥30 LACC resections per year). Data were analysed using Kaplan–Meier curves, logistic regression analysis and Cox‐regression models.

**Results:**

A total of 49 298 patients were diagnosed with colon cancer, of whom 9206 (18.7%) had locally advanced disease. Of these 9206 patients, resection was performed in 8537 with a median age of 71 (interquartile range: 63–78) years. Patients were more likely to undergo laparoscopic procedures in high‐volume hospitals than in low‐volume hospitals (OR = 1.28, 95% CI: 1.12–1.46). No risk differences in anastomotic leakage or postoperative 90‐day mortality were observed according to hospital volume. Five‐year overall survival rates were comparable among high‐, medium‐ and low‐volume hospitals (58.7% vs. 58.0% vs. 60.0%, *p* = 0.62). Hospital volume was not associated with overall survival in multivariable analysis. Independent predictors of worse overall survival included older age, higher American Society of Anaesthesiologists score, emergency/urgent setting, anastomotic leakage, higher pTNM status, involved resection margins and no adjuvant chemotherapy.

**Conclusion:**

Despite the complexity of surgical treatment, hospital volume was not associated with survival in LACC. Hospital volume might be an imperfect surrogate for quality assessment.

**What does this paper add to the literature?**

This study demonstrates no significant association between hospital volume and survival rates after surgical treatment for locally advanced colon cancer, based on comprehensive nationwide data. This highlights that hospital volume alone is insufficient as a quality metric and emphasizes the need for more precise measures to evaluate surgical outcomes.

## INTRODUCTION

Although many screening programmes have been initiated, approximately 10%–20% of patients who present with colorectal cancer are still diagnosed with locally advanced colon cancer (LACC) [1, 2]. In general, LACC refers to clinically staged T3cd (extramural depth ≥5 mm) or T4 tumours (Stage II or Stage III) or to tumours with extensive regional lymph node involvement, usually based on CT scanning [3, 4]. The standard treatment of care with curative intent is complete surgical resection, followed by adjuvant chemotherapy depending on pathological outcome and the patients' condition [5, 6]. However, neoadjuvant chemotherapy (NAC) is gaining popularity, and the recently published update of FOxTROT revealed some benefits of NAC in patients with cT3cd‐cT4 colon cancer [7].

The surgical management of LACC poses a challenge as these tumours, if stage T4b, can extend through the colonic wall into adjacent structures and organs. For complete removal of such tumours, multivisceral resection (MVR) is necessary, and this is accompanied with an increased risk of surgical complications [8, 9]. Nevertheless, achieving negative surgical margins is important, as incomplete margins negatively impact local and distant recurrence rates, leading to reduced overall survival (OS) [10, 11, 12].

In the past decades, several studies have described the association between hospital volume and survival in complex cancer surgery, such as in pancreatic cancer and oesophageal cancer [13, 14]. In colorectal cancer, however, several studies on survival and hospital volume have found conflicting outcomes. This is partly because of various volume definitions, different time periods and accuracy of data reporting [15, 16, 17]. There have been a small number of studies describing the correlation of survival and hospital volume in patients who undergo surgery for locally advanced rectal cancer, also with inconsistent results [16, 18]. For patients with LACC, only limited data are available on the influence of hospital volume on survival [19].

Therefore, the primary aim of this study was to investigate if hospital volume has an influence on long‐term survival following surgery for LACC in a nationwide study in the Netherlands. The secondary objectives were to determine treatment‐related short‐term outcomes, completeness of surgery, perioperative treatment patterns, the incidence of MVR, and 30‐ and 90‐day mortality rates.

## METHOD

### Data collection

For this study, data were retrieved from the nationwide population‐based Netherlands Cancer Registry (NCR). The main notifiers of the NCR are the digital pathology archive and the Dutch hospital discharge register ‘National Basic Register of Hospital Care’ (LBZ). Trained data managers of the NCR routinely extracted patient, tumour and treatment characteristics from medical records. Tumours were classified according to the International Classification of Diseases for Oncology. The TNM staging system was used for stage notification of the primary tumour, according to the edition valid at the time of diagnosis (7th edition for 2010–2016 and the 8th edition for 2017–2019) [20]. Follow‐up of vital status was retrieved by linkage to the Municipal Personal Records Database. There was no ethical approval mandatory for this study according to Dutch law.

### Study population

For this study, LACC was defined as either clinical (c)T4 stage or cN2 stage, without preoperative clinical distant metastases. All patients with LACC who underwent surgical resection between 1 January 2015 and 31 December 2019, in the Netherlands, were included. Patients who only underwent surgical exploration were excluded from analysis because the focus of the study was survival following tumour resection.

The following patient‐ and tumour‐related variables were available: year of diagnosis, gender, age, performance status, American Society of Anaesthesiologists (ASA) classification, clinical and pathological TNM stage, morphology, tumour grade (well/moderately differentiated, poorly differentiated/undifferentiated) and the presence of multiple colorectal cancer tumours and synchronous distant metastases diagnosed during surgery. Available treatment‐related variables were neoadjuvant treatment, adjuvant treatment, type of surgical procedure [ileocecal/(extended) hemicolectomy, transverse resection, (low) anterior/sigmoid, subtotal colectomy/total proctocolectomy], operative setting (elective, emergency/urgent), anastomotic leakage and 30‐ and 90‐day mortality. All hospitals performing colorectal cancer surgery in the Netherlands were included. Hospitals were divided into three mutually exclusive categories: low‐volume hospitals (1–19 LACC resections per year); medium‐volume hospitals (20–29 LACC resections per year); and high‐volume hospitals (≥30 LACC resections per year). The primary outcome was 5‐year OS. Radicality of resection was divided into three categories and designated as follows: complete tumour resection with all margins histologically negative (R0); incomplete tumour resection with a microscopic incomplete resection margin (R1); and tumour resection with a macroscopic incomplete resection margin (R2). Follow‐up was completed on 1 February 2023.

### Statistical analysis

Descriptive statistics were used to provide an overview of baseline patient characteristics. Continuous data were reported as median [interquartile range (IQR)] and categorical data were reported as count (percentage). Patient, tumour, treatment and postoperative outcome were stratified according to low (1–19 LACC resections per year), medium (20–29 LACC resections per year) and high (≥30 LACC resections per) volume hospitals. The chi‐square test was used for comparison of categorical data between the three groups, and Kruskal‐Wallis one‐way ANOVA was used for continuous data.

The association between hospital volume and OS was analysed: survival time was calculated as the time between surgical resection and either the date of death or the last day of follow‐up for patients who were still alive. At the end of follow‐up, patients who were alive were censored in survival analyses. Kaplan–Meier analysis was used to calculate OS. The log rank test was used according to hospital volume. Crude and adjusted Cox regression analyses were performed to evaluate the possible association between hospital volume and 5‐year OS in patients with LACC. To investigate whether survival outcome was dependent on tumour stage, sub‐analyses were performed for patients with stage cT2‐3N2, cT4 and pT4 LACC. Variables from the univariable analyses were entered into a multivariable Cox proportional hazards model when the *p*‐value was <0.1 or when considered clinically relevant. The results are presented as hazard ratios (HRs) with 95% CI. All tests of significance were two‐tailed and a *p*‐value of ≤0.05 was considered statistically significant. Variables with missing data were identified and quantified. For variables with <10% missing data (e.g., ASA classification, tumour grade), we performed complete‐case analyses, which is appropriate when data are missing at random (MAR) and helps maintain statistical power. Variables with significant amounts of missing data (e.g., WHO Performance Status and multiple synchronous tumours) were excluded from multivariable analyses to avoid potential bias and instability in the models. We assessed the patterns of missing data and determined that the data were MAR, meaning that the likelihood of missing data was related to observed data but not to the missing values themselves. Sensitivity analyses, including comparisons of baseline characteristics between patients with and without missing data, were conducted to evaluate the impact of missing data on the results. To adjust comprehensively for potential confounders, a directed acyclic graph, a visual tool that represents the causal relationships between variables, was constructed (Figure S1). Statistical analyses were performed using IBM SPSS Statistics software version 29.0 (SPSS Inc., Chicago, IL, USA).

## RESULTS

From a total of 49 298 patients diagnosed with colon cancer between 2015 and 2019, 9206 (18.7%) were diagnosed with LACC without clinical distant metastases. Of these patients, 3243 (35.2%) had stage cT4 LACC and 1454 (15.8%) had stage cN2 LACC. In 8537 of the patients, a surgical resection was performed. Twenty‐three hospitals were classed as high volume, with a median of 36 (IQR: 30.5–44) LACC resections per year; 19 hospitals were classed as medium volume, with a median of 23 (IQR: 21–26) LACC resections per year; and 38 hospitals were classed as low volume, with a median of 13 (IQR 9.5–17) of LACC resections per year.

The baseline characteristics of the patients who underwent resection are presented in Table 1. The median age was 71 (IQR: 63–78) years and was similar between the three hospital volume groups (*p* = 0.87). From 2015 to 2019, progressively more patients with LACC were operated on in low‐volume hospitals (*p* < 0.001). Neoadjuvant chemotherapy was infrequently administered, and no significant differences regarding the effect of NAC were found between the volume groups (*p* = 0.25). Most patients were operated on in an elective setting (83.0% in a high‐volume hospital, 83.9% in a medium‐volume hospital and 83.2% in a low‐volume hospital; *p* = 0.64). A laparoscopic/robotic approach was the approach most commonly followed in all volume groups, but the incidence of this approach was significantly higher in high‐volume hospitals than in low‐volume hospitals. After correction for potential confounding, this association remained significant (OR = 1.28, 95% CI: 1.12–1.46, after adjustment for gender, age, cTN stage, NAC, MVR, operative setting and year of resection; data not shown).

**TABLE 1:** Baseline characteristics of patients who underwent resection of locally advanced colon cancer between 2015 and 2019: data are stratified according to hospital volume.

	Hospital volume*			
Patient characteristics	Low volume	Medium volume	High volume	*p*‐Value
				*χ* ^2^
Total patients	2186 (25.6)	2419 (28.3)	3932 (46.1)	
Gender				
Male	1070 (48.9)	1195 (49.4)	1948 (49.5)	0.90
Female	1116 (51.1)	1224 (50.6)	1984 (50.5)	
Age (median, IQR)	71 (62–78)	71 (62–79)	71 (63–78)	0.55
Year of diagnosis				
2015	405 (18.5)	545 (22.5)	997 (25.4)	**<0.001**
2016	424 (19.4)	474 (19.6)	926 (23.6)	
2017	432 (19.8)	543 (22.4)	665 (16.9)	
2018	433 (19.8)	486 (20.1)	696 (17.7)	
2019	492 (22.5)	371 (15.3)	648 (16.5)	
WHO performance status				
WHO 0	582 (60.9)	640 (53.3)	1253 (56.7)	**0.02**
WHO 1	294 (30.8)	431 (35.9)	755 (34.2)	
WHO 2	59 (6.2)	106 (8.8)	158 (7.2)	
WHO 3–4	21 (2.2)	23 (1.9)	42 (1.9)	
Unknown (*n* = 4173)				
ASA classification				
ASA 1	241 (12.3)	322 (14.2)	521 (14.3)	**<0.001**
ASA 2	1037 (52.8)	1271 (56.1)	2051 (56.1)	
ASA ≥3	687 (35.0)	672 (29.7)	1082 (29.6)	
Unknown (*n* = 653)				
Multiple synchronous CRC tumours				
Yes	83 (7.0)	76 (5.0)	139 (5.5)	0.07
No	1103 (93.0)	1453 (95.0)	2376 (94.5)	
Unknown (*n* = 3307)				
Morphology				
Adenocarcinoma	1849 (84.6)	2034 (84.1)	3327 (82.9)	0.42
Mucinous	258 (11.8)	290 (12.0)	487 (12.4)	
Signet cell carcinoma	41 (1.9)	57 (2.4)	70 (1.8)	
Other	38 (1.7)	38 (1.6)	48 (1.2)	
Clinical TN stage				
cT2‐T3, cN2	1485 (67.9)	1631 (67.4)	2732 (69.5)	0.19
cT4, cN0‐2	701 (32.1)	788 (32.6)	1200 (30.5)	
Neoadjuvant treatment				
Chemotherapy	59 (2.7)	57 (2.4)	76 (1.9)	0.25
(Chemo)radiation therapy	9 (0.9)	26 (1.1)	31 (0.8)	
No neoadjuvant treatment	2108 (96.4)	2336 (96.6)	3825 (97.3)	
Operative setting				
Elective	1794 (83.2)	2024 (83.9)	3254 (83.0)	0.64
Emergency/urgent	361 (16.8)	389 (16.1)	668 (17.0)	
Unknown (*n* = 47)				
Surgical procedure				
Ileocecal/(extended) hemicolectomy	1394 (63.8)	1519 (62.8)	2524 (64.2)	**<0.001**
Transverse resection	44 (2.0)	46 (1.9)	88 (2.2)	
(Low) anterior/sigmoid	674 (30.8)	808 (33.4)	1242 (31.6)	
Subtotal colectomy/proctocolectomy	59 (2.7)	44 (1.8)	75 (1.9)	
Not otherwise specified	15 (0.7)	2 (0.1)	3 (0.1)	
Surgical approach				
Open	723 (33.6)	770 (31.8)	1202 (30.7)	0.25
Laparoscopic/robotic without conversion	1164 (54.0)	1329 (54.9)	2181 (55.8)	
Laparoscopic/robotic with conversion	267 (12.4)	320 (13.2)	525 (13.4)	
Unknown (*n* = 56)				

*Note*: Data are given as *n* (%) or median (IQR). The significance of the bold p‐values is to indicate statistical significance.

Abbreviations: ASA, American Society of Anesthesiologists; CRC, colorectal cancer; cTN, clinical tumour‐nodal‐stage; IQR, interquartile range; WHO, World Health Organization.

* Hospitals were placed in one of three categories according to the number of low anterior colorectal cancer (LACC) resections they performed each year: low volume (1–19 resections per year), medium volume (20–29 resections per year) or high volume (≥30 resections per year).

Results of pathological and postoperative outcomes are displayed in Table 2. Involvement of circumferential resection margins did not vary significantly between high‐volume, medium‐volume and low‐volume hospitals (94.9%, 95.8% and 95.5%, respectively, *p* = 0.21). Also, anastomotic leakage rates (*p* = 0.14), as well as 30‐day and 90‐day postoperative mortality, did not differ according to hospital volume (*p* = 0.15 and *p* = 0.31, respectively).

**TABLE 2:** Pathological and postoperative outcomes of patients who underwent resection of locally advanced colon cancer between 2015 and 2019: data are stratified according to hospital volume.

	Hospital volume*			
Outcome	Low volume	Medium volume	High volume	*p*‐Value
				*χ* ^2^
Total patients	2186 (25.6)	2419 (28.3)	3932 (46.1)	
Pathological tumour stage				
(y)pT0/x	10 (0.4)	1 (0.0)	11 (0.3)	0.07
(y)pT1	12 (0.5)	15 (0.6)	24 (0.6)	
(y)pT2	71 (3.2)	80 (3.3)	152 (3.9)	
(y)pT3	907 (41.5)	1010 (41.8)	1723 (43.8)	
(y)pT4	1186 (54.3)	1313 (54.3)	2022 (51.4)	
Pathological nodal stage				
(y)pN0	756 (34.6)	816 (34.4)	1352 (34.4)	0.95
(y)pN1	514 (23.5)	591 (23.8)	934 (23.8)	
(y)pN2	916 (41.9)	1012 (41.8)	1646 (41.9)	
Pathological distant metastases				
M0/x	2152 (98.4)	2386 (98.6)	3872 (98.5)	0.84
M+	34 (1.6)	33 (1.4)	60 (1.5)	
Tumour grade				
Well/moderately differentiated	1572 (79.2)	1777 (80.1)	2912 (81.2)	0.20
Poorly differentiated/undifferentiated	412 (20.8)	441 (19.9)	675 (18.8)	
Unknown (*n* = 748)				
Circumferential resection margin				
Microscopic complete (R0)	2075 (94.9)	2318 (95.8)	3754 (95.5)	0.21
Microscopic incomplete (R1)	85 (3.9)	79 (3.3)	151 (3.8)	
Macroscopic incomplete (R2)	26 (1.2)	22 (0.9)	27 (0.7)	
Anastomotic leakage				
No	1847 (84.5)	2019 (83.5)	3318 (84.4)	0.14
Yes, with or without abscess/abscess only	154 (7.0)	132 (5.5)	243 (6.2)	
No anastomosis	185 (8.5)	268 (11.1)	371 (9.4)	
Adjuvant treatment				
Chemotherapy	1055 (48.3)	1194 (49.4)	1916 (48.8)	0.96
(Chemo)radiation therapy	2 (0.1)	2 (0.1)	4 (0.1)	
No adjuvant treatment	1129 (51.6)	1223 (50.6)	2012 (51.2)	
30‐day postoperative mortality				
Yes	76 (3.4‐)	63 (2.6)	104 (2.6)	0.15
No	2111 (96.6)	2356 (97.4)	3829 (97.4)	
90‐day postoperative mortality				
Yes	110 (5.0)	104 (4.3)	166 (4.2)	0.31
No	2076 (94.9)	2315 (95.7)	3766 (95.8)	

*Note*: Data are given as *n* (%).

* Hospitals were placed in one of three categories according to the number of low anterior colorectal cancer (LACC) resections they performed each year: low volume (1–19 resections per year), medium volume (20–29 resections per year) or high volume (≥30 resections per year).

Multivariable logistic regression analysis for 30‐day postoperative mortality showed that in contrast to hospital volume, the factors gender, age, anastomotic leakage, ASA classification and operative setting were independently associated (data not shown). In multivariable logistic regression analysis for 90‐day postoperative mortality, hospital volume was also not an independent risk factor. The variables that were independently associated with 90‐day postoperative mortality were age, anastomotic leakage, ASA classification and operative setting (Table 3).

**TABLE 3:** Evaluation of 90‐day postoperative mortality, using univariable and multivariable logistic regression analyses, among patients who underwent resection for locally advanced colon cancer.

Variables analysed	Univariable logistic regression OR (95% CI)	*p*‐Value	Multivariable logistic regression OR (95% CI)
Hospital volume*			
Low	1		1
Medium	0.85 (0.64–1.12)	0.24	0.94 (0.69–1.29)
High	0.83 (0.65–1.06)	0.14	0.91 (0.69–1.21)
Gender			
Male	1		1
Female	**0.80 (0.65–0.98)**	**0.03**	0.80 (0.63–1.02)
Age	**1.07 (1.06–1.08)**	**<0.001**	**1.05 (1.03–1.06)**
ASA classification			
ASA 1	1		
ASA 2	**5.22 (2.12–12.85)**	**<0.001**	**3.54 (1.42–8.80)**
ASA ≥3	**21.06 (8.65–51.25)**	**<0.001**	**9.90 (4.00–24.51)**
Neoadjuvant chemotherapy			
No	1		1
Yes	0.51 (0.23–1.16)	0.11	0.91 (0.39–2.14)
Operative setting			
Elective	**1**		1
Emergency/urgent	**4.67 (3.78–5.77)**	**<0.001**	**3.32 (2.56–4.30)**
Multivisceral resection			
No	1		1
Yes	1.08 (0.84–1.39)	0.56	0.98 (0.74–1.31)
Anastomotic leakage			
No	1		1
Yes, with or without abscess/abscess only	**5.37 (4.07–7.09)**	**<0.001**	**5.25 (3.82–7.20)**
No anastomosis	**3.72 (2.87–4.83)**	**<0.001**	**1.68 (1.22–2.31)**

*Note*: Statistically significant OR (95% CI) values and *p*‐values are given in bold. ORs were regarded as significant when the 95% CI did not include 1.00.

Abbreviations: ASA, American Society of Anesthesiologists.

* Hospitals were placed in one of three categories according to the number of low anterior colorectal cancer (LACC) resections they performed each year: low volume (1–19 resections per year), medium volume (20–29 resections per year) or high volume (≥30 resections per year).

## SURVIVAL

The median follow‐up time for the patients included in this study was 32.4 (IQR: 17.9–50.8) months.

**FIGURE 1:**
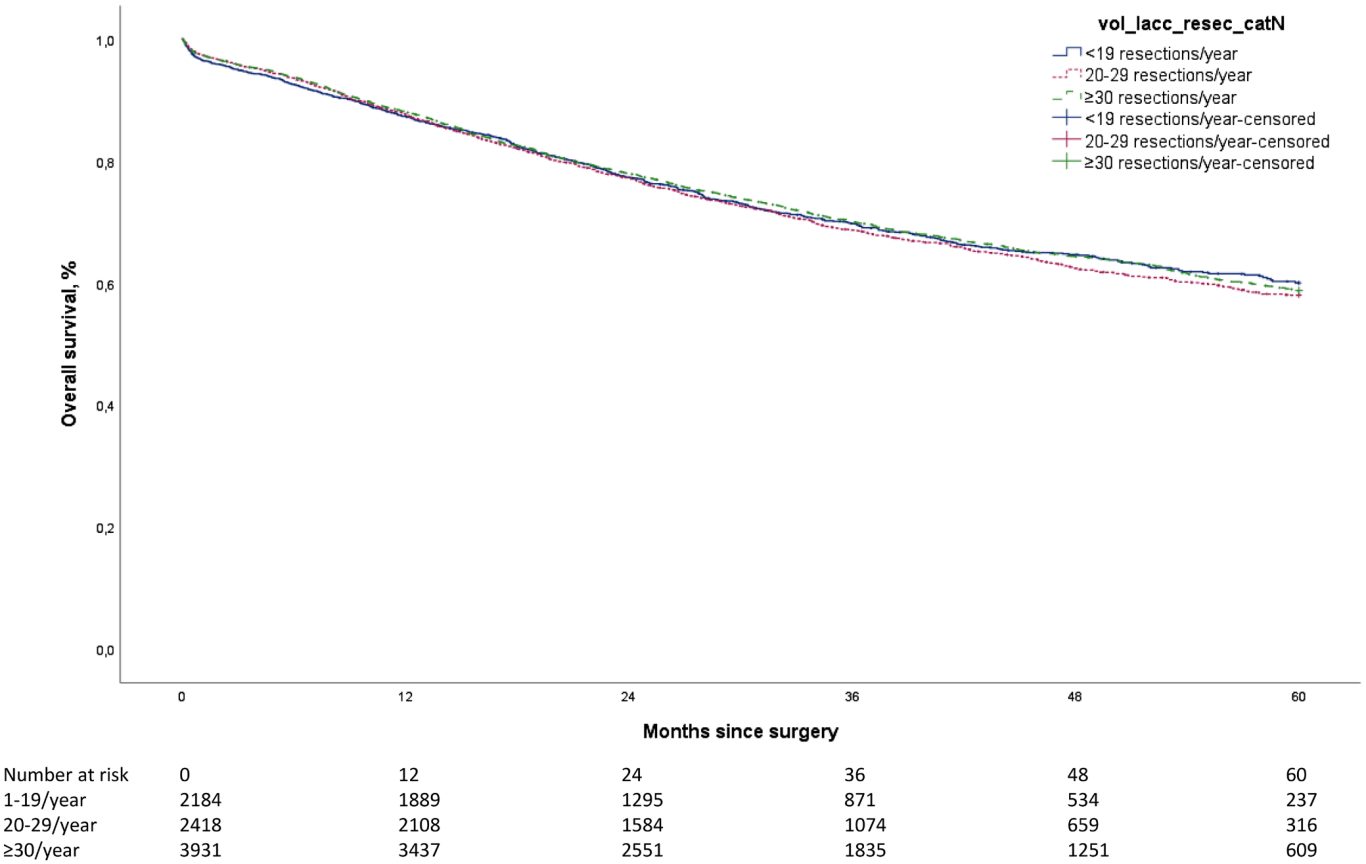
Overall survival of patients with locally advanced colon cancer (LACC) who underwent resection. Survival was stratified according to hospital volume (1–19, 20–29 or ≥30 resections per year). Log rank *p* = 0.62.

**FIGURE 2:**
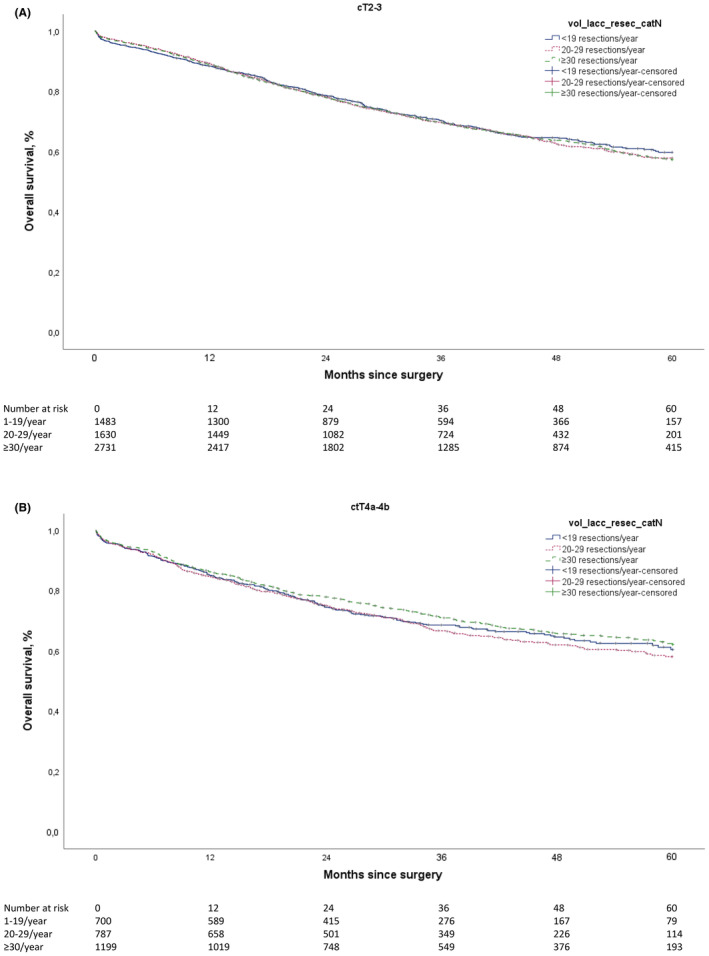
(A) Overall survival for patients with stage cT2‐3N2 (not classified as cT4) locally advanced colon cancer (LACC) who underwent resection. Survival was stratified according to hospital volume (1–19, 20–29 or ≥30 resections per year). Log Rank *p* = 0.60. (B) Overall survival in patients with only cT4 stage LACC who underwent resection. Survival was stratified according to hospital volume (1–19, 20–29 or ≥30 resections per year). Log rank *p* = 0.60.

For patients with LACC, the crude 3‐ and 5‐year observed OS rates were similar for the three hospital volume groups: respectively, 70.0% and 58.7% for high‐volume hospitals, 68.6% and 58.0% for medium‐volume hospitals and 69.7% and 60.0% for low‐volume hospitals (*p* = 0.62; Figure 1). When the analyses were repeated after increasing the threshold for high‐volume hospitals to more than 40 LACC resections per year, similar trends were found for OS (data not shown). In the subgroup of patients with cT2‐3N2 tumours, 5‐year OS (57.3% for high‐volume hospitals vs. 57.9% for medium‐volume hospitals vs. 59.8% for low‐volume hospitals, *p* = 0.60; Figure 2A) was comparable to the 5‐year OS for patients with cT4 tumours (62.2% for high‐volume hospitals vs. 58.1% for medium‐volume hospitals vs. 60.5% for low‐volume hospitals, *p* = 0.60; Figure 2B). In the subgroup of patients with pT4 tumours, no significant difference in 5‐year OS was found between the hospital volume groups (52.3% for high‐volume hospitals, 51.8% for medium‐volume hospitals and 51.0% for low‐volume hospitals, *p* = 0.66; data not shown).

In univariable Cox regression analyses, NAC (HR = 0.57, 95% CI: 0.42–0.76, *p* < 0.001) and adjuvant chemotherapy (HR = 0.45, 95% CI: 0.41–0.49, *p* < 0.001) were identified as significant predictors with a positive impact on survival (Table 4).

**TABLE 4:** Evaluation of overall survival, using univariable and multivariable Cox regression analyses, of all patients who underwent resection of locally advanced colon cancer.

Variables analysed	Univariable analysis Hazard ratio (95% CI)	*p* Value	Multivariable analysis Hazard ratio (95% CI)
Hospital volume*			
Low	1		
Medium	1.04 (0.94–1.15)	0.44	1.07 (0.96–1.20)
High	1.00 (0.91–1.10)	0.99	1.00 (0.90–1.11)
Gender			
Male	1		
Female	0.99 (0.91–1.06)	0.69	0.93 (0.85–1.01)
Age	**1.05 (1.05–1.05)**	**<0.001**	**1.02 (1.02–1.03)**
ASA classification			
ASA 1	1		1
ASA 2	**1.88 (1.61–2.20)**	**<0.001**	**1.43 (1.21–1.69)**
ASA ≥3	**4.05 (3.46–4.74)**	**<0.001**	**2.19 (1.84–2.62)**
Neoadjuvant chemotherapy			
No	1		1
Yes	**0.57 (0.42–0.76)**	**<0.001**	**0.70 (0.50–0.97)**
Operative setting			
Elective	1		1
Emergency/urgent	**2.12 (1.95–2.31)**	**<0.001**	**1.45 (1.30–1.62)**
Multivisceral resection			
No	1		
Yes	1.04 (0.95–1.14)	0.37	**1.08 (0.97–1.20)**
Anastomotic leakage			
No	1		1
Yes, with or without abscess/abscess only	**1.71 (1.49–1.96)**	**<0.001**	**1.34 (1.34–1.82)**
No anastomosis	**2.01 (1.80–2.23)**	**<0.001**	**1.31 (1.15–1.49)**
Pathological tumour stage			
(y)T0/x‐(y)T2	1		1
(y)T3	**1.65 (1.28–2.13)**	**<0.001**	1.31 (1.00–1.73)
(y)T4	**2.68 (2.08–3.44)**	**<0.001**	**2.26 (1.71–3.00)**
Pathological nodal stage			
(y)N0	1		1
(y)N1	**1.53 (1.38–1.70)**	**<0.001**	**1.83 (1.63–2.07)**
(y)N2	**1.75 (1.60–1.92)**	**<0.001**	**3.29 (2.93–3.70)**
Pathological distant metastases			
M0/x	1		1
M+	**3.17 (2.54–3.95)**	**<0.001**	**2.05 (1.60–2.63)**
Tumour grade			
Well/moderately differentiated	1		1
Poorly differentiated/undifferentiated	**1.76 (1.61–1.92)**	**<0.001**	**1.40 (1.27–1.54)**
Resection margins			
Microscopic complete (R0)	1		1
Microscopic incomplete (R1)	**2.10 (1.79–2.45)**	**<0.001**	**1.53 (1.27–1.85)**
Macroscopic incomplete (R2)	**4.40 (3.38–5.72)**	**<0.001**	**3.09 (2.29–4.17)**
Adjuvant chemotherapy			
No	1		1
Yes	**0.45 (0.41–0.49)**	**<0.001**	**0.46 (0.41–0.51)**

*Note*: Statistically significant hazard ratio (95% CI) values and *p*‐values are given in bold. Hazard ratios were regarded as significant when the 95% CI did not include 1.00.

Abbreviation: ASA, American Society of Anesthesiologists.

* Hospitals were placed in one of three categories according to the number of low anterior colorectal cancer (LACC) resections they performed each year: low volume (1–19 resections per year), medium volume (20–29 resections per year) or high volume (≥30 resections per year).

In the multivariable Cox regression model, hospital volume was not associated with survival, whereas higher age, higher ASA classification level, emergency/urgent procedures, anastomotic leakage, and no anastomosis, pT4 stage, pN1‐2 stage, metastases found during surgery, poorly differentiated/undifferentiated tumour grade and R status were independently associated with poorer survival (Table 4). The statistically significant associations between NAC and adjuvant chemotherapy and OS remained after correction for confounders (HR = 0.70, 95% CI: 0.50–0.97 for NAC and HR = 0.46, 95% CI: 0.41–0.51 for adjuvant chemotherapy).

Sensitivity analyses confirmed that missing data did not significantly affect the outcomes. Comparisons of baseline characteristics showed no significant differences between patients with complete data and those with missing data, supporting the assumption that data were MAR. The primary results of the analyses remained consistent, indicating that the findings are robust despite the missing data.

## DISCUSSION

This nationwide population‐based study evaluated the impact of hospital volume on long‐term survival in patients who underwent resection of LACC between 2015 and 2019. No differences in 3‐ or 5‐year OS were found for patients with LACC who underwent a resection in low‐volume, medium‐volume, or high‐volume centers. Also, multivariable analysis showed that hospital volume did not have an impact on OS. Independent prognostic factors for OS were found to be age, ASA score, NAC, operative setting, anastomotic leakage, pTNM status, tumour grade, circumferential resection margins and adjuvant chemotherapy.

Conflicting results have been published regarding the relationship between hospital volume and long‐term outcome in colorectal cancer [15, 18, 21, 22]. Historical studies often used data based on resections with a high laparotomy rate that were performed by non‐specialized surgeons, used heterogeneous definitions of high and low volume, and omitted adjustments for important prognostic factors [17]. A multicentre German study analysed a cohort of patients with locally advanced rectal cancer who were treated with pre‐ or postoperative 5‐fluorouracil‐based chemoradiotherapy between 1995 and 2002 and found a significantly positive association between high hospital volume and 10‐year OS. Yet, the study did not account for potential confounding patient‐, tumour‐ and treatment‐related risk factors, such as age, gender, pathological stage and (neo)adjuvant treatment [23]. By contrast, a recent study from the Netherlands reported no effect of hospital volume in patients who underwent surgery for cT4 rectal cancer after adjusting for relevant confounders, such as neoadjuvant therapy [16]. Neoadjuvant therapy has become standard practice for patients with locally advanced rectal cancer, although there is still marked variation in whether it is administered across hospitals in the Netherlands [24].

Studies focusing specifically on hospital volume and LACC are rather scarce. A recent study using data from the Dutch colorectal audit described short‐term outcomes of patients with cT4 and/or pT4 LACC. Decreased R0 rates were reported among patients with pT4(a–b) stage LACC in comparison with patients with lower pT stages. There were low rates of MVR, with a significantly lower proportion of R0 resections than of standard resections, and no significant differences between hospital volume groups. The hospitals were categorized into low‐volume (≤5 procedures per year) and high‐volume (>5 procedures per year) for patients with cT4 and/or pT4 stage LACC who underwent MVR [25]. Similarly, in the present study there were no differences in R0 rates between the volume groups, although the definition of LACC was slightly different (cT4 and/or cN2 disease). In a recent Swedish study, involving 5241 patients with LACC defined as pT4, a positive association was reported between high‐volume hospitals and OS [19]. All‐cause and colon cancer‐specific mortality were significantly lower in high‐volume hospitals, defined as >19 resections/year. The authors reported 3‐year OS of 68% in high‐volume hospitals, compared with 60% in medium‐volume and 58% in low‐volume hospitals (*p* < 0.001). Comparison with the results of the present study is difficult because of different definitions of LACC and hospital volume. In the present study, 3‐year OS in high‐volume hospitals was almost identical (70%), but the survival in low‐volume hospitals was much higher than in the Swedish study (69.7% vs. 58%). The discrepancy between the findings of the Swedish study and those of the present study might be explained by the difference in definition of LACC and/or hospital volumes, but also differences in confounders included in multivariable analyses might have led to the divergent results.

In the present study, there were no differences in the frequency of administering NAC among the three hospital volume groups, and only a small number of patients in each group received NAC. At the time of the study, neoadjuvant treatment was not considered standard practice, because data from the FOxTROT or other studies were not yet available and implemented in the guidelines [7]. Multivariable Cox regression analysis showed a possible survival benefit of NAC in the population of the present study, although this should be interpreted with caution because of the risk of residual bias. Similarly, a previous Dutch study demonstrated the potential benefit of downstaging by NAC [26]. Several prospective studies published after the promising results of the FOxTROT trial describe good feasibility and tolerability of administration of NAC in LACC [3, 27, 28, 29]. Recently, superior 2‐year disease control and a 28% lower recurrence rate were demonstrated in the FOxTROT trial [7]. Similarly, in the PRODIGE 22 trial, significant tumour regression was shown, without a difference in pathological stage or survival, although the study was underpowered [27]. Neoadjuvant chemotherapy as a treatment option for LACC is evolving, and further research to refine the optimal indication based on clinical staging, dose and sequence of NAC in LACC remains to be established.

A recent study using data from the Dutch Cancer Registry demonstrated a higher likelihood of MVR if patients with colon cancer were more recently diagnosed in, or were treated in, high‐volume hospitals [9]. However, in accordance with previous studies, many of the patients did not undergo MVR in the present study [9, 12]. The preoperative decision of whether to perform MVR is difficult and is mostly made intra‐operatively [9, 12, 30]. Extended resections are associated with increased morbidity and postoperative complication rates of around 22%–36% [12]. Despite its drawbacks, the oncological principle of *en bloc* resection should be followed if there is a clinical suspicion of tumour adherence.

Although many patients still undergo a laparotomy for LACC, laparoscopic surgery in LACC has been considered safe in selected patients with lower morbidity. Presently, a gradual increase in the incidence of laparoscopic surgery for LACC is observed, as demonstrated by the present study. In a cohort study including 86,680 patients from the USA, it was reported that minimally invasive surgery in Stage 3 colon cancer was associated with earlier initiation of adjuvant systemic therapy and survival benefit compared with open surgery [31]. Another recent large cohort study from the USA similarly revealed improved short‐term outcomes and longer OS in favour of minimally invasive surgery [32]. This suggests that appropriately selected patients with LACC should preferably undergo a minimally invasive resection.

This large nationwide cohort provides unique long‐term data on the influence of hospital volume on 5‐year OS in patients who underwent resection of LACC in daily practice. Over the past decade, advancements in surgical techniques, perioperative care and multimodality treatment have substantially contributed to enhancements in the quality of care in the treatment of LACC. Probably, these advances have also been adopted by lower‐volume hospitals, resulting in overall improvements in the complex and demanding surgical treatment of LACC. Nonetheless, hospital volume could be considered as a surrogate marker because of the lack of more appropriate available variables to measure the quality of surgical treatment. Although we did not find a correlation between hospital volume and outcome, this does not mean that individual experience and volume of surgeons is not important. Individual surgeon volume is probably a better marker for quality assurance, and the caseload of a surgeon appears to interact more clearly with survival than hospital volume does [17]. Surgical technique is most likely an important determinant of patient outcomes [33, 34]. Intra‐operative video registration, with analysis using an objective validated competency assessment tool, in which technical skill is scored could potentially demonstrate whether surgical technical skills are associated with long‐term survival following complex colon cancer surgery [33, 34, 35]. Thereby, these tools that assess performance facilitate comparison of surgeons and hospitals and can be incorporated in teaching programmes to improve ultimately patient outcomes [35, 36]. Future studies on assessing individual surgical quality might become increasingly important if we aim for further improvements in the quality of colorectal surgery.

The results from this study should be interpreted with caution as there is a chance of bias and residual confounding because of the observational nature of the study. The reliability of preoperative CT staging in colon cancer is moderate, as there is a weak correlation between the radiological T/N stages and the actual pathological stages. In Dutch hospitals, nearly half of cT1‐2 tumours are under staged, while many cT3‐4 and cN1‐2 tumours are over staged [37]. The inability to accurately classify patients into high‐ and low‐risk groups may have also influenced the outcomes in this study. Similarly, the limited accuracy of CT staging was evident in the FOxTROT study, in which 24% of patients had pT3N0 tumours and did not meet standard criteria for adjuvant therapy [7]. Future improvements in imaging techniques or the development of new radiological staging systems, such as the CT TDV scoring system, which includes high‐risk features on baseline CT, such as extramural venous invasion and tumour deposits, could help address this issue [38]. Unfortunately, the NCR also lacks data on variables that are likely to be associated with the choice of surgical approach and that may also independently influence survival (such as prior surgical history, date and location of recurrences). Also, data on pathological items, such as vascular invasion or location of incomplete resections, which could offer valuable insights into patient outcomes, are missing. Such data might have provided crucial information for better patient stratification and understanding of OS. Furthermore, it is uncertain if substantially increasing the volume threshold for a high‐volume hospital or using volume as a continuous value could have led to identification of a clear, optimal volume threshold for improved outcomes. While both methods have their merits, we believe that our categorical approach strikes a balance between statistical robustness and clinical interpretability. However, hospital volume alone is probably not an appropriate measure of quality. Also, information on subdivision in pT4a stage or pT4b stage was mainly missing and could therefore not be studied.

## CONCLUSION

The results of the present study do not confirm the presence of a relationship between hospital volume and outcome in LACC surgery. No differences were demonstrated in postoperative mortality and long‐term survival rates among patients with LACC who underwent surgery in Dutch hospitals that were stratified for low, medium and high volumes. However, this finding does not indicate that quality of surgery does not influence short‐term outcomes and survival. Hospital volume might function as an imperfect surrogate for quality assurance, and further studies are needed with accurate parameters and tools based on surgical quality assessment.

## AUTHOR CONTRIBUTIONS

**L. C. F. de Nes:** Conceptualization; investigation; writing – original draft; writing – review and editing; formal analysis; methodology; project administration. **P. J. Tanis:** Writing – review and editing; supervision. **R. H. Verhoeven:** Writing – review and editing; supervision. **J. H. W. de Wilt:** Conceptualization; writing – review and editing; supervision; formal analysis. **P. A. J. Vissers:** Conceptualization; writing – review and editing; supervision; formal analysis; resources; project administration.

## FUNDING INFORMATION

There was no funding for this study.

## CONFLICT OF INTEREST STATEMENT

P. J. Tanis received research grants from Allergan (LifeCell) and the Dutch Cancer Society unrelated to the submitted work. R. H. Verhoeven received research grants from Roche and Bristol‐Myers Squibb unrelated to the submitted work. J. H. W. de Wilt reports research grants from ZonMW, the Dutch Cancer Society, Medtronic and Roche unrelated to the submitted work. The other authors have no conflicts of interest or financial ties to disclose.

## ETHICS STATEMENT

There was no ethical approval and patient consent mandatory for this study according to Dutch law.

## PERMISSION TO REPRODUCE MATERIAL FROM OTHER SOURCES

There was no use of previously copyrighted material.

## Supporting information


Figure S1.

## Data Availability

The data that support the findings of this study are available from the corresponding author upon reasonable request.
