# Supplementary material for: Impact of hospital volume on survival in patients with locally advanced colon cancer – A Dutch population‐based study
Source: Colorectal Dis. 2025 Jan 26;27(2):e17288. doi: 10.1111/codi.17288 (PMC11873530; doi:10.1111/codi.17288)
Supplement: Supplementary file 1 — Figure S1. [file CODI-27-0-s001.docx]

**
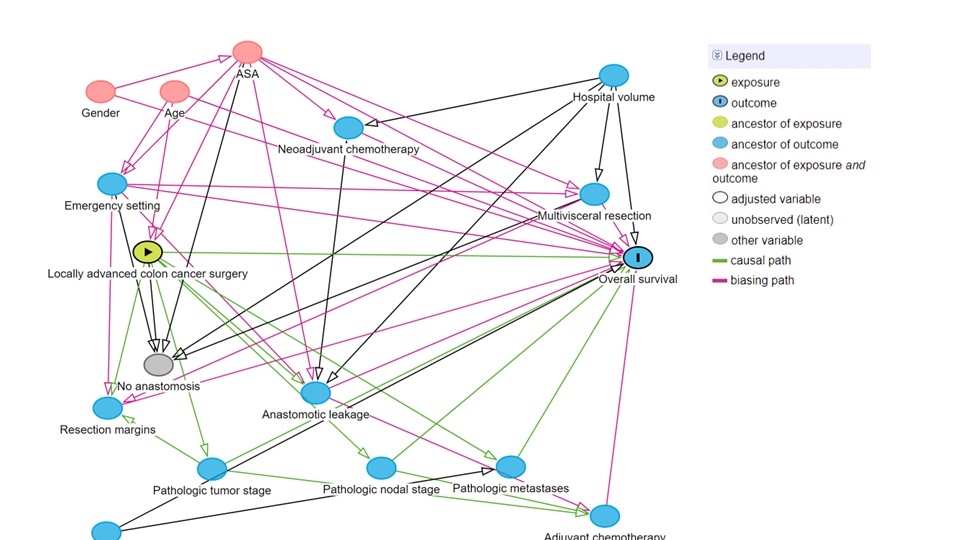
**

**Supplementary fig. 1. Directed acyclic graph of the assumed relationships between hospital volume, possible mediators and/or confounders and survival after locally advanced colon cancer resections**

*Please use colors in the figure*
